# High pressure structural behaviour of 5,5′-bitetrazole-1,1′-diolate based energetic materials: a comparative study from first principles calculations†

**DOI:** 10.1039/d0ra04782a

**Published:** 2020-07-02

**Authors:** B. Moses Abraham

**Affiliations:** Advanced Centre of Research in High Energy Materials (ACRHEM), University of Hyderabad Prof. C. R. Rao Road, Gachibowli Hyderabad-500046 Telangana India mosesabrahamb@gmail.com

## Abstract

Pressure on the scale of gigapascals can cause incredible variations in the physicochemical and detonation characteristics of energetic materials. As a continuation of our earlier work (B. Moses Abraham, *et al.*, *Phys. Chem. Chem. Phys.*, 2018, **20**, 29693–29707), here we report the high pressure structural and vibrational properties of 5,5′-bitetrazole-1,1′-diolate based energetic ionic salts *via* dispersion-corrected density functional theory calculations. Remarkably, these energetic materials exhibit anisotropic behavior along three crystallographic directions with progressing pressure; especially, the maximum and minimum reduction in volume is observed for HA-BTO and TKX-50, respectively. The large bulk modulus of TKX-50 (28.64) indicates its hard nature when compared to other BTO-based energetic salts. The effect of pressure on hydrogen bonded D–H⋯A energetic materials induces spectral shift (lengthening/shortening) in the donor group (D–H) of the stretching vibrations and is widely recognized as the signature of hydrogen bonding. We observed unusual contraction of the D–H bond under compression due to the short range repulsive forces encountered by the H atom while the molecule attempts to stabilize. The Hirshfeld surface analysis highlights the pressure induced stabilization of HA-BTO due to increased N⋯H/H⋯N and O⋯H/H⋯O close contact of hydrogen bond acceptors and donors. These studies provide theoretical guidance as a function of pressure, on how the micro-structures and intermolecular interactions can tune macroscopic properties to enhance the energetic performance.

## Abstract 2

We report the high pressure structural and vibrational properties of 5,5′-bitetrazole-1,1′-diolate based energetic ionic salts *via* dispersion-corrected density functional theory calculations.

## Graphical abstract

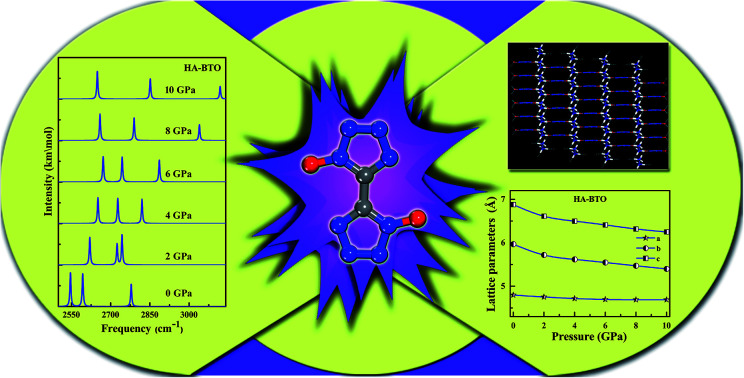

## 1 Introduction

Energetic materials include a wide range of substances with a large amount of stored chemical energy that can react to release massive amounts of power upon external stimulation. Typically, when energetic materials detonate, the shock wave produces a temperature of 3500 K and the internal pressure soars up to 500 000 times that of Earth’s atmosphere.^1,2^ The crystal density and detonation characteristics of energetic molecular crystals that exclusively depend on structure and the type of interactions, can undergo remarkable variations under extreme conditions.^3,4^ In this regard, it is important to understand and identify the microscopic response of explosives to temperature and pressure conditions. Moreover, the subtle structural difference when subjected to external pressure can show strong implications in the properties as well as stability of the molecules. However, the extreme sensitivity of energetic materials to an accidental stimulus such as shock, impact, light or friction can trigger undesired and unintended detonation, which is a great challenge in developing advanced high energy density materials (HEDM) with desired properties.

The application of pressure can provide efficient crystal packing of the structures of energetic materials, thereby tuning the two most crucial properties: energy and safety. Since the detonation velocity of an explosive is directly proportional to the packing density,^5^ the effect of pressure can increase the compactness by reducing the free volume in the crystal and hence increasing the energy of an explosive.^6^ On the other hand, the van der Waals (vdW) interactions and hydrogen bonding increase with ascending pressure, leading to a stable structure; nevertheless, excess compression may cause disadvantageous hot spot formation and molecular degradation, and subsequently contribute to a lower safety. The effect of pressure may also induce variations in electrostatic interactions, vdW forces, hydrogen bonding networks, π–π stacking and other effects, leading to new molecular rearrangements and reorientations, thereby tuning the crystal structure symmetry.^7–9^ However, as a pervasive intermolecular interaction, the precise knowledge of hydrogen bonding is very essential because of its importance in understanding the dynamics of chemical systems, function and structural stability.^11^ These hydrogen bonding networks among the molecules of crystalline materials are significant as they can provide fundamental insight into the behavior and properties of elements and chemicals, and help in applications such as tunable sensitivity of energetic materials, hydrogen storage, pharmaceuticals, *etc.* Especially, the contribution of hydrogen bonding to energetic materials is absolutely outstanding owing to their improvised detonation characteristics. As the physico-chemical properties of molecular crystals are closely connected to their structure, the compression of hydrogen bonded energetic materials is subject to extensive research to determine the structure–property relationships. Therefore, the corresponding molecular level investigation under an applied pressure could provide basic information to the unusual properties of energetic crystals.

Hydrogen bonded systems can be studied using state-of-the-art X-ray diffraction techniques. However, the X-rays and neutrons are scattered by different constituents of the atom, which may induce a significant difference in electron density centroids and neutron scattering density. Therefore, X-ray crystallography at synchrotron sources underestimates the D–H covalent bond lengths^10^ and is also unable to detect hydrogen bonds *via* a diamond anvil cell due to a limited set of Bragg intensity data. While, hydrogen bonded networks, especially as a function of pressure, can be effectively studied using vibrational spectroscopy. The infrared active antisymmetric, and the Raman active symmetric, modes of D–H stretching vibrations can be analyzed constructively using this approach. Perhaps the major drawback is the presence of very intense and broad stretching modes due to overlap of overtones, combination bands and interference from cascading Fermi resonances.^12^ This can be attributed to the enhancement in anharmonicity as the strength of hydrogen bonds increases under pressure thereby making it difficult to determine the exact peak positions. On the other hand, density functional theory (DFT) simulations have been increasingly used to study intermolecular interactions, especially hydrogen bonding in a wide range of pressures.^13^ The standard DFT techniques using pseudopotential approaches *via* local density (LDA) or generalized gradient (GGA) approximations are mostly employed for analyzing hydrogen bonded solids. Nevertheless, the success and failure of conventional DFT functionals to account for vdW interactions is in debate,^14–19^ but this can be overcome by including a wide range of newly developed dispersion-corrected functionals. Moreover, these computational studies can provide valuable information to avoid accidents during experimentation and manufacturing processes. The pioneering scientist Dr Betsy M. Rice and her co-workers from the Army Research Laboratory, United States, comprehensively investigated the effect of semiempirical corrections in standard DFT to account for vdW interactions at ambient, as well as hydrostatic, pressures for a series of energetic materials including RDX, HMX, CL20, PETN, TATB, TNT and FOX-7.^20–22^ Their results provide an accurate description for describing intermolecular interactions in energetic molecular crystals, while standard DFT severely underestimates the crystallographic lattice parameters. Subsequently, they have highlighted the importance of dispersion corrected methods for various high nitrogen energetic salts^23^ and compression studies of RDX.^24,25^ Therefore, theoretical simulations have become an indispensable tool by playing an ever-increasing role in unravelling the chemical and physical properties of energetic materials both in ambient and extreme conditions.

As a continuation of our previous work,^27^ here we discuss pressure induced structural variations, vibrational spectra and Hirshfeld surface analysis of 5,5′-bitetrazole-1,1′-diolate based energetic ionic salts (EIS). The variations in the bond shapes of the donor group (D–H) for these energetic materials under increasing pressure represents a strong influence of the hydrogen bonding D–H stretching modes in vibrational dynamics. The Hirshfeld surface analysis provides a clear picture of how the intermolecular interactions in these BTO energetic salts varies as a function of pressure.

## 2 Computational methods

The electronic structures of various energetic salts were investigated using DFT *via* Cambridge Series of Total Energy Package (CASTEP)^26^ within the framework of the plane-wave pseudopotential approach. The two prominent parameters for converging crystal structures – the sampling of *k*-points in reciprocal space and the value of kinetic energy cutoff (size of the basis set) – were chosen according to our previous studies.^27^ The generalized gradient approximation (GGA) developed by Perdew, Burke and Ernzerhof^28^ was implemented to describe the exchange–correlation potentials. The ultrasoft and norm-conserving pseudopotentials were used to calculate the structural properties and zone-center IR spectra, respectively, as a function of pressure up to 10 GPa. The convergence criterion for self-consistent iterations and the maximal ionic Hellmann–Feynman forces were set to 5.0 × 10^−6^ eV per atom and 0.01 eV Å^−1^, respectively. In order to maintain consistency with our previously developed methodology,^27^ we incorporated Grimme’s D2 dispersion correction method to treat the vdW interactions. This approach has already proved to be most successful in describing the structural properties of EIS.^27^ Density functional perturbation theory (DFPT) is used to calculate the vibrational spectra of the studied BTO based energetic salts *via* a linear response method.

The Hirshfeld surfaces^29^ and 2D fingerprint plots^30^ for visualization of intermolecular interactions as a function of pressure up to 10 GPa, was estimated using CrystalExplorer 3.1 (ref. 31) software. The Hirshfeld surfaces are constructed by screening space in the crystal into sections where the electron distribution of a sum of sphere-shaped atoms for the molecule (*i.e.* the pro-molecule) dominates the corresponding sum over the crystal (*i.e.* the pro-crystal). This method was developed for partitioning the crystal electron density into molecular fragments by defining a molecular weight function:1
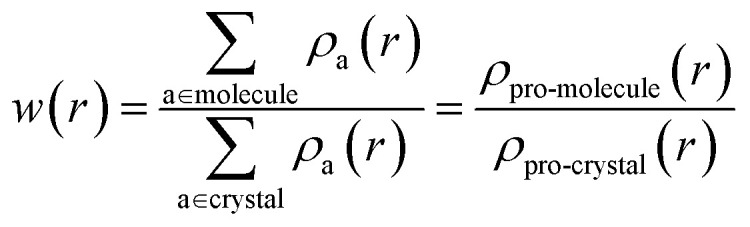
Here, *ρ*_a_(*r*) is a spherically average atomic electron density function centred on nucleus a.^32^ Hirshfeld surfaces are produced through the partitioning of space within a crystal where the ratio of pro-molecule to pro-crystal electron densities is equal to 0.5, resulting in continuous non-overlapping surfaces.^29,33^

The strength of the interactions can be described by *d*_norm_ (normalised contact distance):2
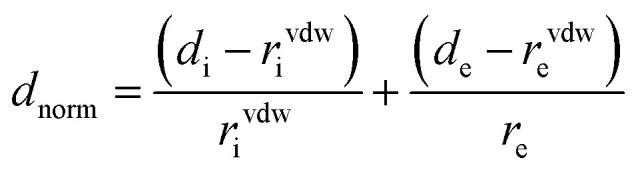
where *r*_i_ and *r*_e_ denote the vdW radii of two atoms inside and outside the Hirshfeld surfaces, respectively; *d*_i_ and *d*_e_ represent the internal and external separations from the nearest atoms, respectively. The 3D *d*_norm_ surface is used to identify close intermolecular contacts, in which the positive and negative values denote the intermolecular contacts that are longer and shorter than the vdW separations, respectively.

## 3 Results and discussion

**Fig. 1:**
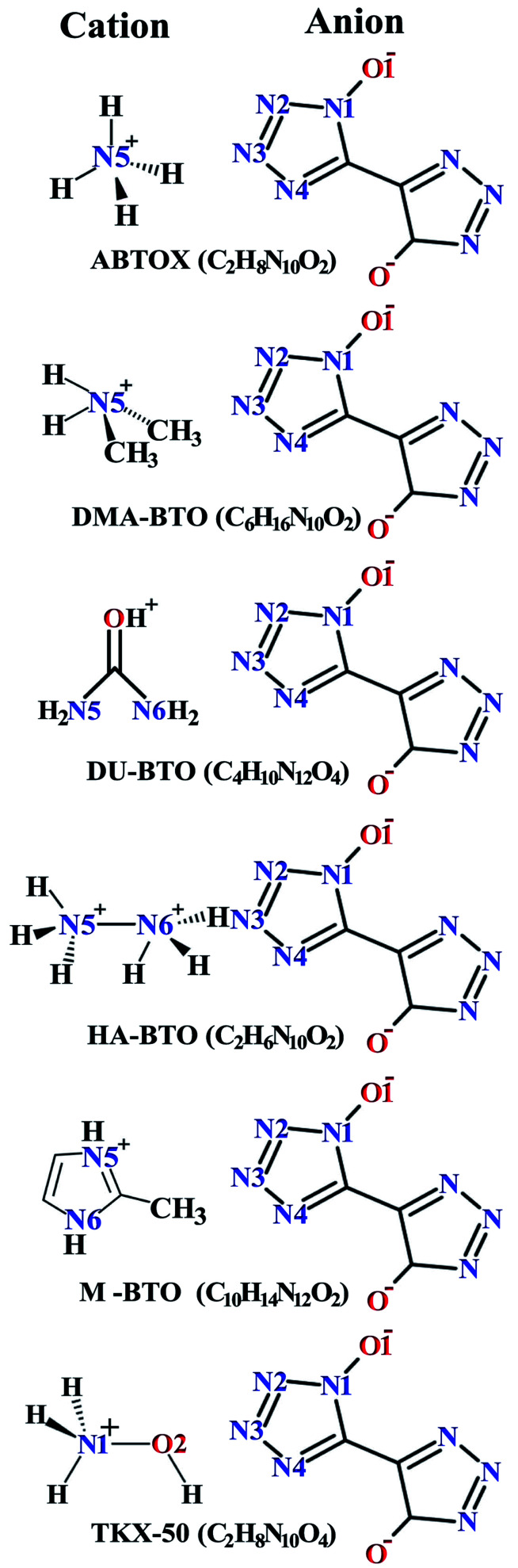
Chemical structures of 5,5′-bitetrazole-1,1′-diolate based energetic salts.

**Fig. 2:**
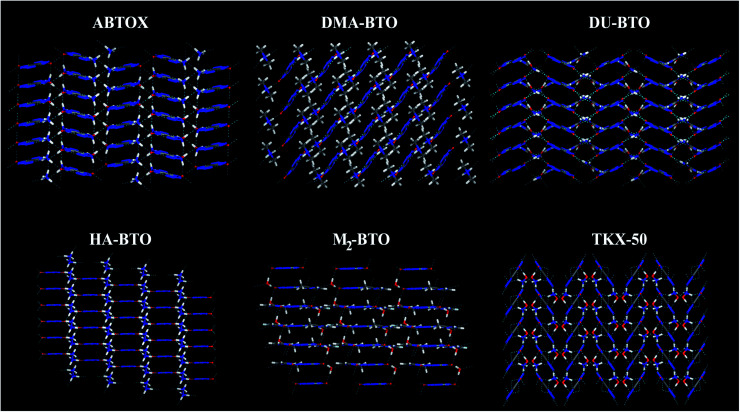
Molecular stacking structures of six BTO-based energetic materials. Here C, H, N and O atoms are represented in grey, white, blue and red, respectively. Green dashed lines represent intermolecular hydrogen bonding networks.

Crystallographic data is fundamental for investigating hydrogen bonded structures. As mentioned in our earlier work,^27^ the structures of M_2_-BTO,^34^ HA-BTO^35^ and DMA-BTO^36^ crystallize in the same triclinic space group *P*1̄, while TKX-50,^37^ DU-BTO^38^ and HA-BTO^39^ stabilize in the monoclinic space groups *P*2_1_/*c*, *P*2_1_/*n* and *P*2_1_/*c*, respectively. The chemical structures of 5,5′-bitetrazole-1,1′-diolate based energetic salts are shown in Fig. 1. Based on electronegative atoms and acidic hydrogen atoms, the target molecule/ion is divided into a number of hydrogen bond acceptor and donor units, each acceptor and donor unit includes one or more electronegative atom(s) or acidic hydrogen atom(s), respectively. These energetic molecular crystals consisting of azoles with a high content of oxygen and nitrogen anions and cations possess more O–H and N–H bonds, exhibiting extensive hydrogen bonding networks as shown in Fig. 2. These hydrogen bond donor–acceptor units are connected by pairing to form a layer-by-layer assembly of molecules with strong hydrogen bonds that can provide fascinating material characteristics. The crystal packing in HA-BTO and M_2_-BTO molecules contain face-to-face π–π stacking. Such crystal structures can be found in insensitive energetic materials like TATB^40,41^ with face-to-face π–π interactions that can exhibit exceptional physicochemical and detonation properties.^42^ The structures of ABTOX and DU-BTO are dominated by wavelike stacking, which is similar to that of insensitive explosive FOX-7.^43^ In the case of TKX-50, the planar bistetrazole anions are V-shaped and face-to-face π-stacked along crystallographic *b*- and *a*-directions, respectively.

**Fig. 3:**
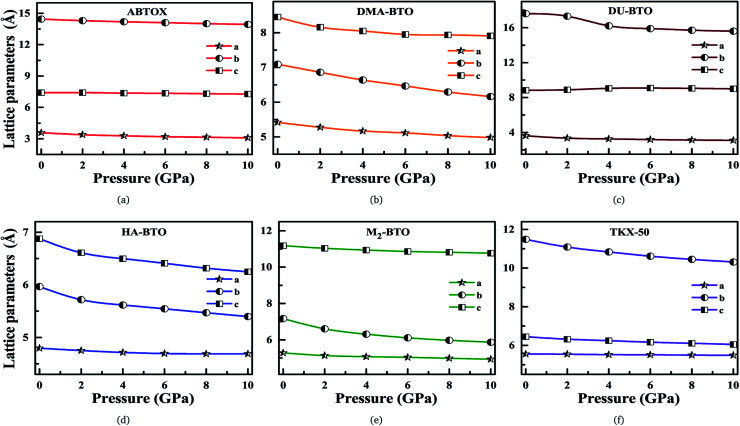
Calculated unit cell parameters (*a*, *b*, *c*) for (a) ABTOX, (b) DMA-BTO, (c) DU-BTO, (d) HA-BTO, (e) M_2_-BTO and (f) TKX-50 as a function of pressure up to 10 GPa.

Our fundamental objective in the present work is to understand the behavior of hydrogen bonding in the studied energetic salts as a function of pressure. To analyze the effect of pressure on the crystal structure of these energetic materials, we performed a comprehensive analysis of the crystal structures at various pressures up to 10 GPa in a step size of 2 GPa at 0 K. The calculated pressure dependent unit cell parameters (*a*, *b* and *c*) of various EIS are presented in Fig. 3. Remarkably, these energetic materials show significant anisotropic nature along three crystallographic directions as a function of pressure. The lattice parameters *a*, *b* and *c* of ABTOX are found to reduce by 13.67%, 3.58% and 1.78%, respectively. For a clear understanding, the normalized lattice parameters as a function of pressure are presented in Fig. S1,† which shows that the axial compressibility of DMA-BTO along the *a*, *b* and *c* axes are reduced by 8.04%, 13.12% and 6.42%, respectively. In the case of DU-BTO, the lattice parameters *a* and *b* compressed to 14.56% and 11.45%, while the *c*-axis is relaxed to 0.17% at 10 GPa. Typically, the application of pressure compresses most of the material in all directions, except for a few systems which expand along a particular direction when subjected to pressure.^44–46^ Several metal–organic frameworks and inorganic compounds are found to show negative linear compressibility,^47–49^ whereas very few organic crystals including TKX-50 (ref. 50) have slight negative compressibility along the *a*-axis. Recently, the structural phase transition and negative linear compressibility has been observed in energetic silver azide along the *a*-direction as a function of pressure.^51^ For TKX-50, the lattice constants *a*, *b* and *c* are reduced by 0.06, 1.17 and 0.39, respectively at 10 GPa, which indicates that the compressibility along the *b*-axis is much greater than that of the *a* and *c*-axes. The low compressibility of the *a*-axis is due to strong interactions between molecules as the intermolecular distances are shorter in the *a*-direction compared to other axes. Moreover, the cations and anions in the crystal structure of TKX-50 are linked together by hydrogen bond networks (N–H⋯O, O–H⋯O) along the *a*-axis. Further, from Fig. S1(f),† the lattice parameters *a*, *b* and *c* of TKX-50 are compressed to 1.2%, 10.24% and 6.11%, respectively, at 10 GPa. This calculated axial compressibility of TKX-50 is in good agreement with previous experimental and theoretical results.^50,52^ The large scale molecular dynamics simulations were also performed on TKX-50 to study its mechanical response to shocks along the three crystallographic directions, and the corresponding Hugoniot elastic limits show impact sensitivity with the [110] direction being the least sensitive.^53^

**Fig. 4:**
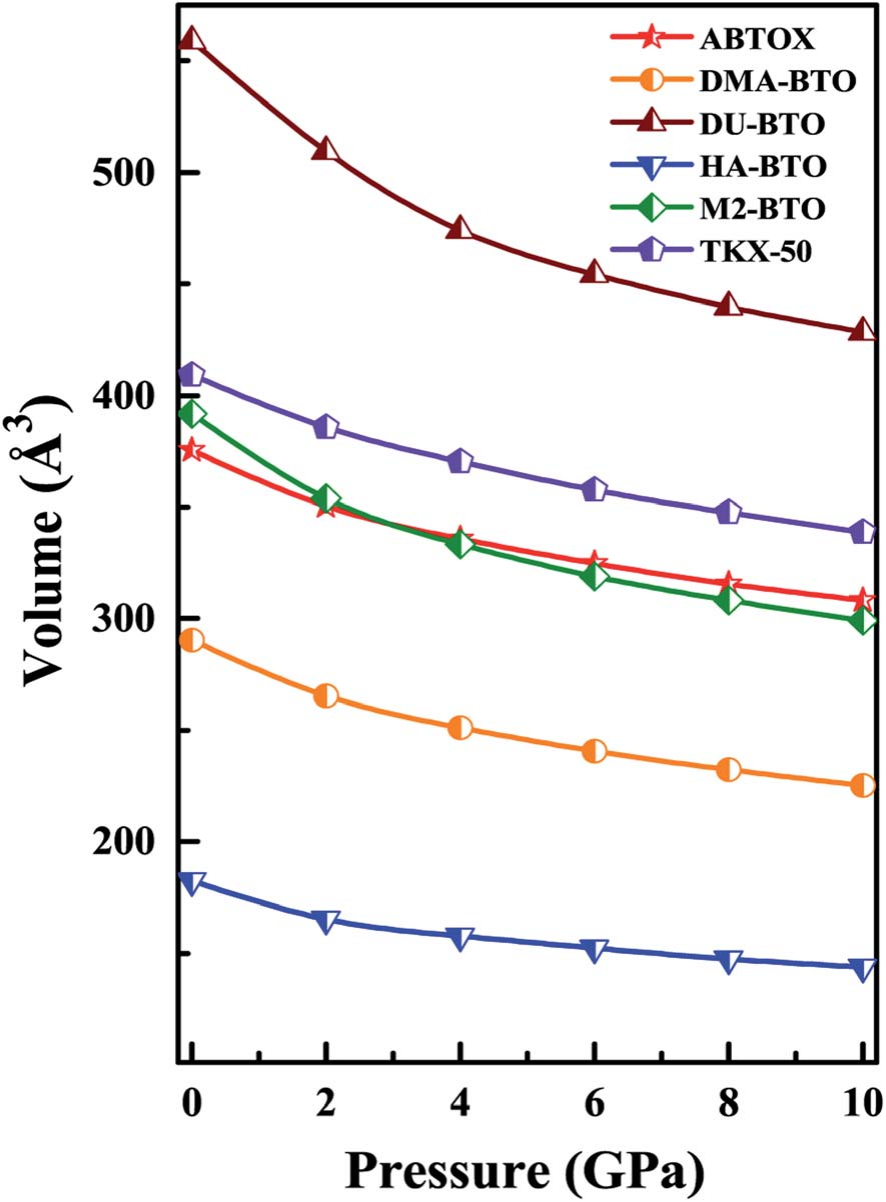
The calculated volume as a function of pressure for the six BTO based energetic materials.

Density is one of the key parameters of energetic materials and a value of >1.80 g cm^−3^ is mandatory to achieve high energy density materials.^54^ The calculated volume as a function of pressure for the studied energetic materials is shown in Fig. 4. In the case of ABTOX, the volume is reduced from 375.3 Å to 308.0 Å when pressure reaches 10 GPa, which in turn increases the density from 1.80 g cm^−3^ to 2.20 g cm^−3^. For DMA-BTO and DU-BTO, the volume is reduced by 22.3% and 23.3%, respectively, and the corresponding density is enhanced by 28.8% and 30.45%. Typically, the application of pressure compresses the crystal structure by facilitating efficient crystal packing, thereby improving the crystal density of a material. The HA-BTO structure with ambient volume and density of 182.5 Å and 1.83 g cm^−3^ shows an equal decrement and increment of 21.25% as pressure progresses to 10 GPa. In the case of M_2_-BTO and TKX-50, their volumes, 391.8 Å and 409.1 Å, decrease to 299.2 Å and 338.7 Å, respectively, and the corresponding densities are increased by 30.9% and 20.7%. The normalized volume and density under the whole pressure range are presented in Fig. S2.† It can be clearly seen that the maximum reduction in volume is observed for HA-BTO; while the minimum is for TKX-50, which implies that the former is more sensitive to pressure. Subsequently, similar behavior is also observed in density where HA-BTO and TKX-50 show maximum and minimum enhancement in density, respectively. Further, the bulk modulus (*B*_0_) and its pressure derivatives 
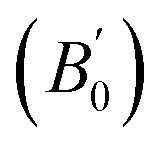
 are computed *via* the third order Birch Murnaghan equation of state^55^ by fitting the pressure–volume data (see Table 1). The *B*_0_
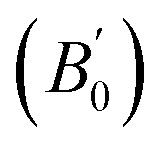
 values are found to be 22.29 (9.03), 16.84 (6.98), 12.17 (9.45), 12.44 (9.21), 12.76 (8.66), 28.64 GPa (6.67) for ABTOX, DMA-BTO, DU-BTO, HA-BTO, M_2_-BTO and TKX-50, respectively. Overall, the minimum bulk modulus corresponds to DU-BTO, while the maximum is obtained for TKX-50, which indicates that the latter is harder than the other studied energetic salts. Further, these energetic salts are as compressible as molecular solids like aurophilic gold(i) iodide^56^ and metal–organic frameworks;^57^ while the calculated bulk modulus of TKX-50 is significantly greater than the other conventional secondary explosives such as PETN-I (12.3 GPa),^58^ β-HMX (15.7 GPa),^59^ α-RDX (13.9 GPa)^58^ and nitromethane (8.3 GPa).^60,61^

**Table 1:** Calculated equilibrium volume (*V*_0_), bulk modulus (*B*_0_) and its pressure derivatives 
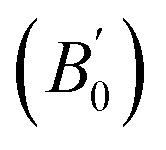
 obtained by fitting the pressure–volume data to the third order Birch Murnaghan equation of state

System	*V*_0_ (Å^3^)	*B*_0_ (GPa)	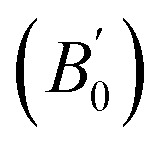
ABTOX	375.30	22.29	9.03
DMA-BTO	290.34	16.84	6.98
DU-BTO	558.78	12.17	9.45
HA-BTO	182.56	12.44	9.21
M_2_-BTO	391.88	12.76	8.66
TKX-50	409.11	28.64	6.67

Intra- and inter-molecular bonding as a function of pressure shows drastic variations, which play a predominant role in understanding the crystal stability of these energetic materials. The calculated intramolecular D–H bond length, intermolecular H⋯A and D⋯A distances, and D–H⋯N bond angles under ascending pressure up to 10 GPa for these BTO-based EIS are shown in Fig. S3.† For ABTOX, the D–H bond lengths decrease except for N5–H5D, which first increases and then decreases under pressure. The N5–H5A and N5–H5B bonds of DMA-BTO show the opposite trend. In the case of HA-BTO (TKX-50), the N5–H5B (O2–H2 and N5–H5A) distance increases, while the other bonds decrease under pressure. In contrast, the D–H bond lengths of M_2_-BTO remain unchanged with the same motif throughout the pressure range. It should be noted that the decrease of D–H bond length indicates the strengthening of the bonds, thereby making it more difficult to release the hydrogen atom. Typically, the covalent D–H distance should enlarge under pressure, while H⋯A interactions reduce to minimize repulsive forces, which leads to an overall shortening of the D⋯A distance. The H⋯A distances decrease with increasing pressure, except for the H5B⋯N4, H2C⋯N1 and H5C⋯N3 of DMA-BTO, DU-BTO and HA-BTO, respectively. For ABTOX, the average H⋯A distance at 0 GPa (1.90 Å) and 10 GPa (1.75 Å) is smaller than the sum of the vdW radii of H and N/O (∼2.7 Å). Similarly, the average intermolecular H⋯A distance for DMA-BTO, DU-BTO, HA-BTO, TKX-50 and M_2_-BTO at 10 GPa is around 1.91, 1.92, 1.86, 1.96 and 1.76, respectively, representing short H⋯A contacts in the latter under pressure. Further, the pressure induced reduction in the D⋯A interactions vary according to the intermolecular distances, which is larger for longer contacts than the smaller ones. While the bond angles of all six energetic salts are less sensitive and barely change with pressure.

### 3.1 Vibrational properties

**Fig. 5:**
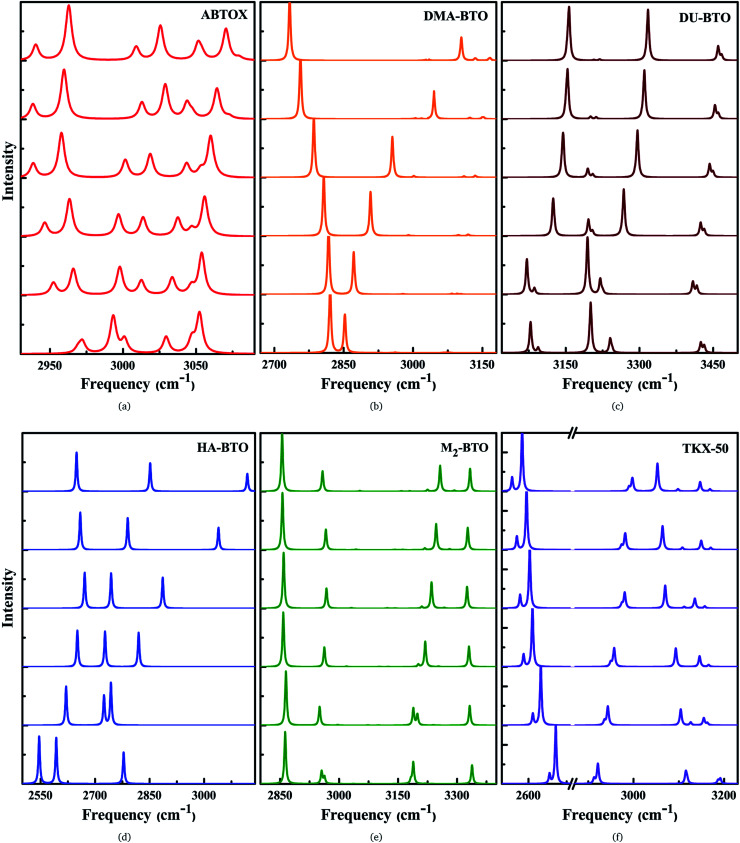
Calculated pressure induced IR spectra of high frequency symmetric/asymmetric stretching vibrations for (a) ABTOX, (b) DMA-BTO, (c) DU-BTO, (d) HA-BTO, (e) M_2_-BTO and (f) TKX-50 as a function of pressure up to 10 GPa.

Vibrational spectroscopy is a powerful technique to measure the lattice and molecular vibrations in the Brillouin zone center, and can detect the variations in molecular configuration or structural distortion *via* the observation of soft modes and/or band splitting. The theoretical IR spectra for the studied energetic salts at ambient pressure are already reported in our earlier work^27^ and the corresponding spectra as a function of pressure up to 10 GPa in step sizes of 2 GPa are shown in Fig. 5(a–f) and S4.† For all the studied energetic salts, the frequency of lattice modes, especially below 400 cm^−1^ (far IR region) are found to increase, indicating pressure induced hardening of lattice modes. In the case of ABTOX and DMA-BTO, the NH_4_ scissoring (1695–1729 cm^−1^) and CH_3_ wagging (1386–1413 cm^−1^) modes show a blue shift as a function of pressure, respectively. The NH_2_ and OH rocking modes of DU-BTO located around 1043–1054 cm^−1^ show a large red shift under compression. For HA-BTO, the frequency of modes at 1098 (NH_2_ rocking, N–N stretching and N=N twisting), 1552 and 1641 cm^−1^ (NH_2_ scissoring and NH rocking) show a red shift; while 1205 (NH_3_ wagging and ring stretching), 1320 (NH_2_ wagging, C=N and N=O stretching) and 1386 cm^−1^ (NH_2_ wagging, C–N stretching) modes exhibit a blue shift under pressure. In the case of TKX-50, the effect of pressure leads to a blue shift in the majority of peaks located between 859–1586 cm^−1^.

The IR symmetric/asymmetric stretching vibrations under an applied pressure are very important to understand the variations in hydrogen bonding networks. Therefore, these vibrations in the high frequency region belonging to CH_2_, CH_3_, NH_2_, NH_3_, NH_4_, OH and OH_2_ symmetric/asymmetric stretching modes are presented in Fig. 5(a–f). In the case of TKX-50, the asymmetric stretching of NH and OH (2671) cm^−1^, and symmetric stretching of NH_2_ (3108 cm^−1^) modes show a red shift; while the NH and OH symmetric stretching mode (2911 cm^−1^) exhibits the opposite trend as a function of pressure. Interestingly, the NH_2_ asymmetric stretching mode located around 3194 cm^−1^ (see Fig. 5f) shows a slight reversal under pressure from red to a blue shift. Similar behavior is also observed in the case of HA-BTO and DU-BTO, where the NH_2_ symmetric stretching vibrations of HA-BTO (2777 cm^−1^) and DU-BTO (3240 cm^−1^) exhibit a gradual reversal under pressure. These variations in the hydrogen bonding networks between cation and anion (N–H⋯O and N–H⋯N) restrict further elongation of covalent N–H bonds due to repulsions from the surrounding neighbors in the crystal confinement.^62^ It should be noted that these changes in corresponding vibrations do not seem to manifest a phase transition, which is consistent with earlier studies of TKX-50;^63^ where spectral changes of TKX-50 were observed in a very limited number of vibrations over a broad pressure range, indicating structural adjustment rather than phase transition.

Typically, the effect of pressure on hydrogen bonded systems D–H⋯A (A and D denote acceptor and donor, respectively) induce spectral shift (lengthening/shortening) in the donor group (D–H) of the stretching vibrations, which is widely recognized as the signature of hydrogen bonding.^64^ The D–H bond lengthening can be visualized as “a consequence of a stabilizing interaction”, generally known as electrostatic interactions,^65,66^ in which the negative A pulls the positive H towards itself resulting in a red shift due to the weakening of the D–H bond. This is clearly observed in DMA-BTO where the asymmetric stretching mode located at 2820 cm^−1^ shows a red shift as a function of pressure. As mentioned earlier, similar behavior is also observed in the case of TKX-50 for the modes located at 2671 and 3108 cm^−1^. This effect not only increases the intensity but also enhances the interaction of the D–H stretching band in the vibrational spectra. On the other hand, there exists an unusual contraction of the D–H bond under compression, leading to a blue shift in the stretching frequency. The shortening of the D–H bond can be attributed to the short range repulsive forces encountered by the H atom while attempting stabilization. Such features are observed in the NH_2_ asymmetric stretching vibrations of ABTOX located at 3052 cm^−1^, which shows a blue shift under pressure. DMA-BTO also exhibits such a strong blue shift in the high frequency stretching mode located at 2852 cm^−1^. The OH and ring CH stretching mode (3182 cm^−1^) of M_2_-BTO show a similar trend as a function of pressure. The pressure induced lengthening and/or shortening of the D–H donor group may induce similar variations in the hydrogen bond acceptor without any major fundamental distinctions between the mechanism of formation. Overall, these variations in the bond shapes of the donor group (D–H) under pressure represent the strong influence of hydrogen bonding D–H stretching modes in vibrational dynamics.

### 3.2 Hirshfeld surface analysis

**Fig. 6:**
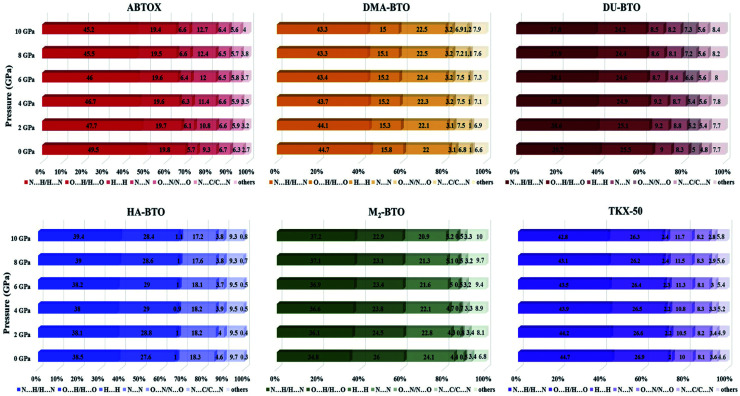
The percentage contributions with ascending pressure of different intermolecular interactions to the Hirshfeld surface area of various BTO-based energetic materials.

The Hirshfeld surface area is a general method of determining molecular size, which resembles the molecular weight. It explores the explicit intra- and inter-molecular atom–atom close interactions and describes the region and types of intercontacts in the crystal packing, thereby providing a fraction of measurable value to the close contacts.^30,67^ Here, the CrystalExplorer software^31^ that includes asphericity, globularity, surface area and the enclosed volume of the Hirshfeld surface of each molecule is used to calculate the intermolecular interactions present in the system. The percentage contributions as a function of pressure for different intermolecular contacts to the Hirshfeld surface area of various energetic salts are shown in Fig. 6. As mentioned in our earlier report,^27^ the N⋯H/H⋯N and O⋯H/H⋯O interactions are the majority contacts in the six energetic materials, which play a predominant role in stabilizing the crystal. The relative contributions of N⋯H/H⋯N and O⋯H/H⋯O interactions in ABTOX decrease from 49.5% to 45.2% and from 19.8% to 19.4%, respectively, under ascending pressure; while, the contributions of H⋯ H and N⋯N increase from 5.7% to 6.6% and from 9.3% to 12.7%, respectively. In the case of DU-BTO, the N⋯H/H⋯N and O⋯H/H⋯O contacts reduced by 1.4% and 0.8%, respectively; whereas, H⋯H and N⋯C/C⋯N interactions increased by 0.5% and 1.3%, respectively. Similar behavior is also observed in the case of DU-BTO (TKX-50) where the N⋯H/H⋯N and O⋯H/H⋯O interactions reduced by 1.9% and 1.3% (1.9% and 0.6%) under an applied pressure.

Recently, we investigated the pressure induced variations in the Hirshfeld surface area of energetic material 3,6-dihydrazino-*s*-tetrazine (DHT)^68^ and found that the strong intermolecular contacts N⋯H/H⋯N increase from 58.3% to 59.3%, while the H⋯H interactions decrease from 26.3% to 19.5%. We also reported the strengthening of hydrogen bonding by showing an elongation in the covalent N–H bond lengths as pressure progresses, which is in accord with the red shift of NH/NH_2_ stretching vibrations. A similar trend is also observed in the case of TKX-50 as a function of pressure up to 30 GPa.^69^ In our study, HA-BTO (M_2_-BTO) was also found to exhibit similar behavior, where the N⋯H/H⋯N contacts increase from 38.5% to 39.4% (from 34.8% to 37.2%); while the O⋯H/H⋯O interactions are enhanced by 0.8% (reduced by 3.1%). It should be noted that the close contact of hydrogen bond acceptors and donors, especially N⋯H/H⋯N and O⋯H/H⋯O interactions, are mainly responsible for hydrogen bonding, and the enhancement of these contacts in HA-BTO under the whole pressure range represent the strengthening of hydrogen bonding. The O⋯O interactions that usually increase the probability of unexpected explosions do not show any impact as pressure progresses.^70–72^ The H⋯H interaction exhibits a maximum increment and decrement of 0.9% and 3.2% for ABTOX and M_2_-BTO, respectively. The significant contribution of N⋯N contacts in HA-BTO show the highest reduction from 18.3% to 17.2%, while ABTOX exhibits the largest enhancement from 9.3% to 12.7% over the whole pressure range. These results highlight the pressure induced stabilization of HA-BTO due to the increase in N⋯H/H⋯N and O⋯H/H⋯O close contact of hydrogen bond acceptors and donors.

## 4 Conclusions

In summary, dispersion-corrected DFT calculations were performed with increasing pressure up to 10 GPa to understand the structural and vibrational variations in the 5,5′-bitetrazole-1,1′-diolate based EIS. The linear compressibility curves show the anisotropic nature along three crystallographic directions, while the *P*–*V* data show that the volume is most and least compressible for HA-BTO and TKX-50, respectively. The predicted high bulk modulus reveals that TKX-50 is harder than other BTO-based energetic materials as well as conventional secondary explosives. The donor group (D–H) of the stretching vibrations in hydrogen bonded D–H⋯A energetic systems show spectral shift (shortening/lengthening) under an applied pressure, representing the signature of hydrogen bonding. We could observe D–H bond lengthening as a consequence of a stabilizing interaction, leading to a red shift in the stretching frequency. The percentage contributions with ascending pressure for different intermolecular interactions to the Hirshfeld surfaces demonstrate the stabilization of HA-BTO due to an increase in N⋯H/H⋯N and O⋯H/H⋯O close contact of hydrogen bond acceptors and donors.

## Conflicts of interest

The author declares no conflicts of interest.

## Supplementary Material

RA-010-D0RA04782A-s001
