# Supplementary material for: High pressure structural behaviour of 5,5′-bitetrazole-1,1′-diolate based energetic materials: a comparative study from first principles calculations
Source: RSC Adv. 2020 Jul 2;10(42):24867–76. doi: 10.1039/d0ra04782a (PMC9055204; doi:10.1039/d0ra04782a)
Supplement: RA-010-D0RA04782A-s001 [file RA-010-D0RA04782A-s001.pdf]

Electronic Supplementary Material (ESI) for RSC Advances.
This journal is © The Royal Society of Chemistry 2020

Supplementary Information

High pressure structural behaviour of 5, 5′-bitetrazole-1,1′-diolate based energetic materials: a comparative study from first principles calculations

B. Moses Abraham*

Advanced Centre of Research in High Energy Materials (ACRHEM), University of Hyderabad, Prof. C. R. Rao Road, Gachibowli, Hyderabad-500046, Telangana, India.

*E-mail: mosesabrahamb@gmail.com

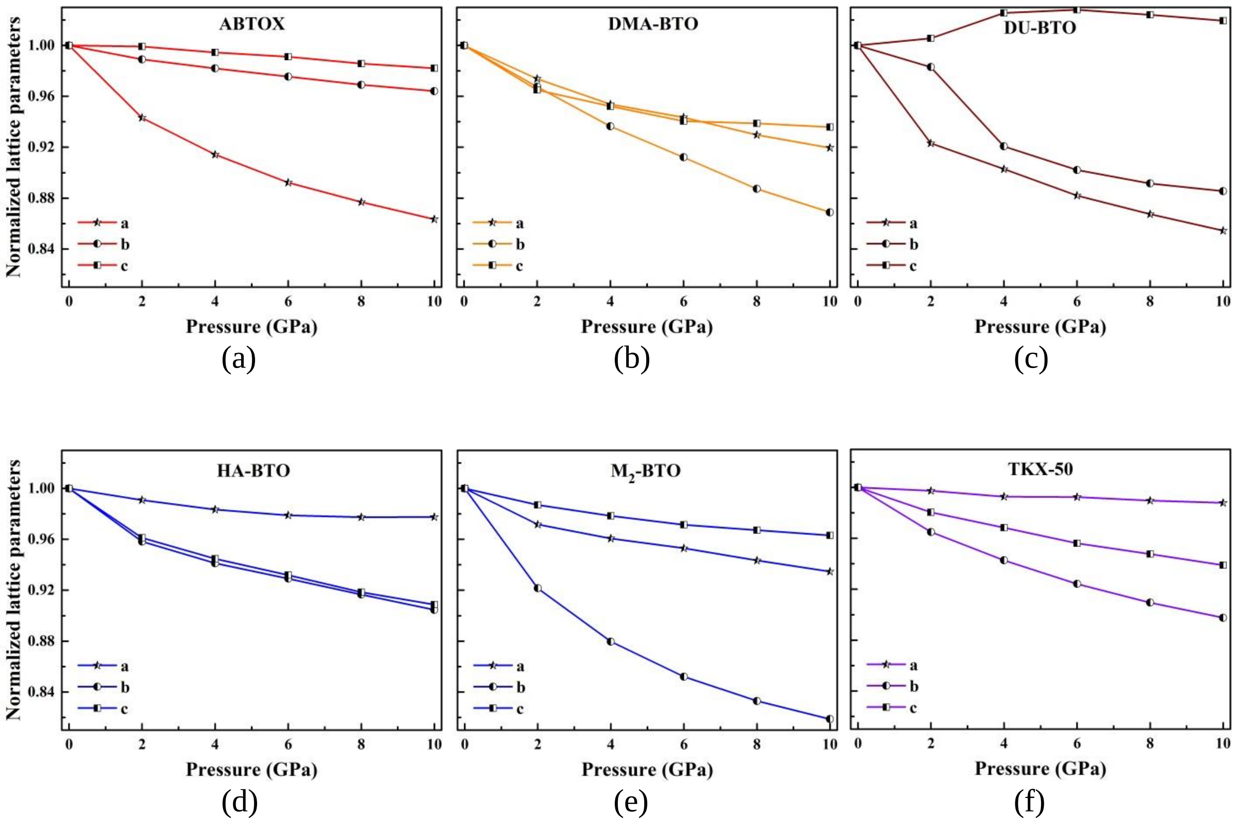

Fig. S1. Normalized lattice parameters under progressing pressure for (a) ABTOX, (b) DMA-BTO, (c) DU-BTO, (d) HA-BTO, (e) M_2-BTO and (f) TKX-50.

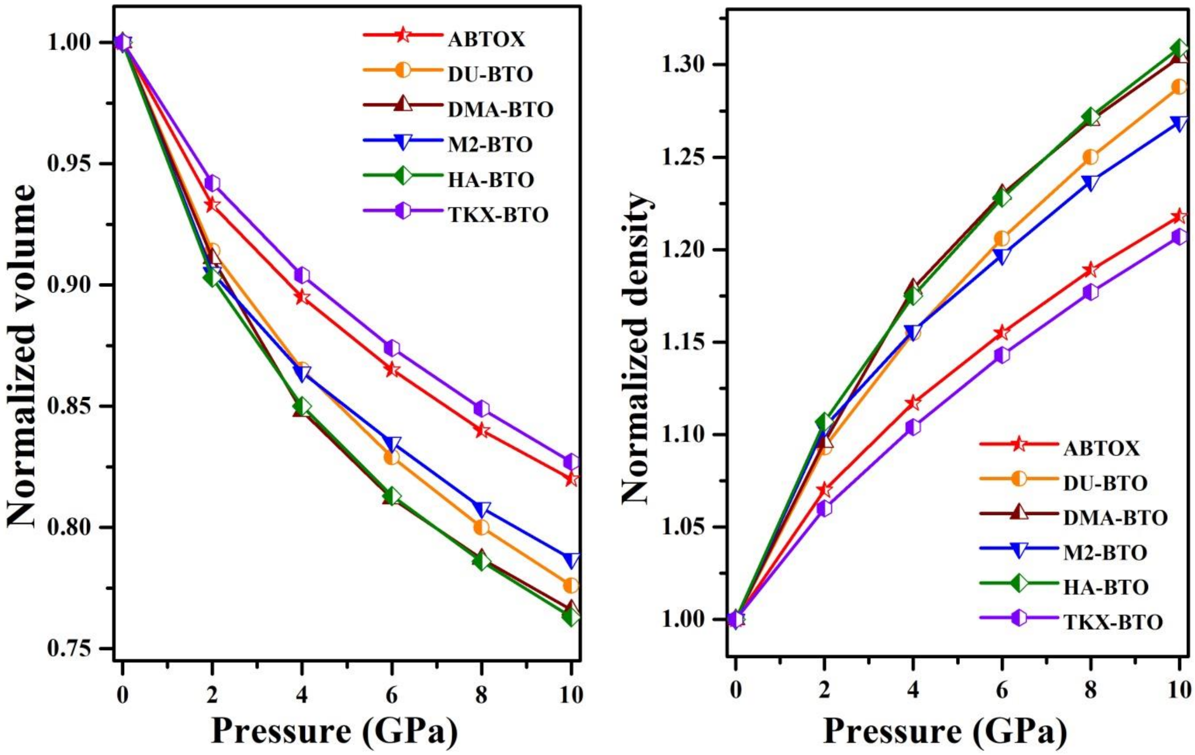

(a) (b)

Fig. S2. Normalized (a) volume and (b) density as a function of pressure for the studied six BTO- based energetic materials.

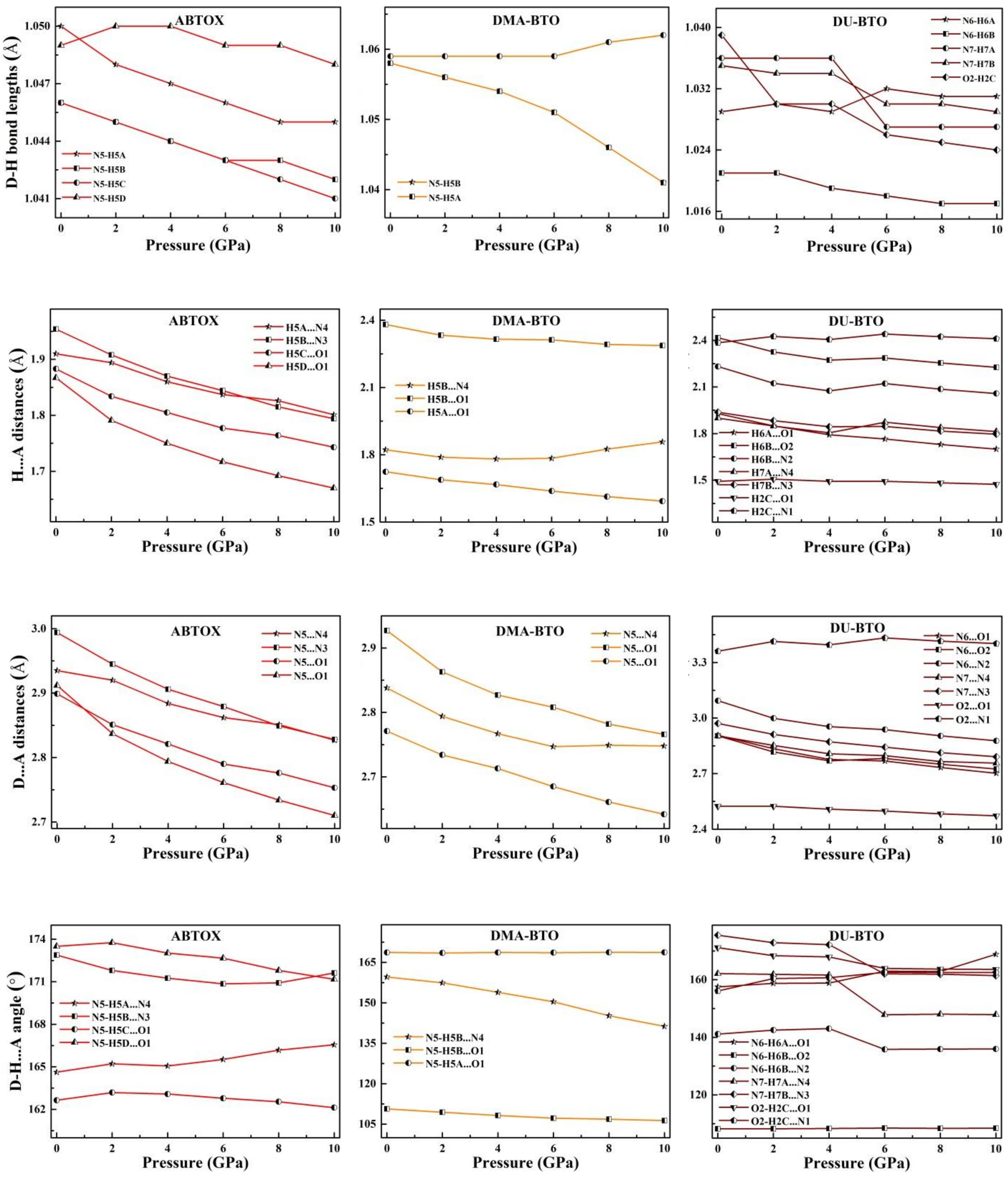
ABTOX
DMA-BTO
DU-BTO
D-H bond lengths (Å)
N5-H5A
N5-H5B
N5-H5C
N5-H5D
N6-H6A
N6-H6B
N7-H7A
N7-H7B
O2-H2C
H...A distances (Å)
H5A...N4
H5B...N3
H5C...O1
H5D...O1
H5B...N4
H5B...O1
H5A...O1
H6A...O1
H6B...O2
H6B...N2
H7A...N4
H7B...N3
H2C...O1
H2C...N1
D...A distances (Å)
N5...N4
N5...N3
N5...O1
N6...O1
N6...O2
N6...N2
N7...N4
N7...N3
O2...O1
O2...N1
D-H...A angle (°)
N5-H5A...N4
N5-H5B...N3
N5-H5C...O1
N5-H5D...O1
N5-H5B...N4
N5-H5B...O1
N5-H5A...O1
N6-H6A...O1
N6-H6B...O2
N6-H6B...N2
N7-H7A...N4
N7-H7B...N3
O2-H2C...O1
O2-H2C...N1
Pressure (GPa)

(a) (b) (c)

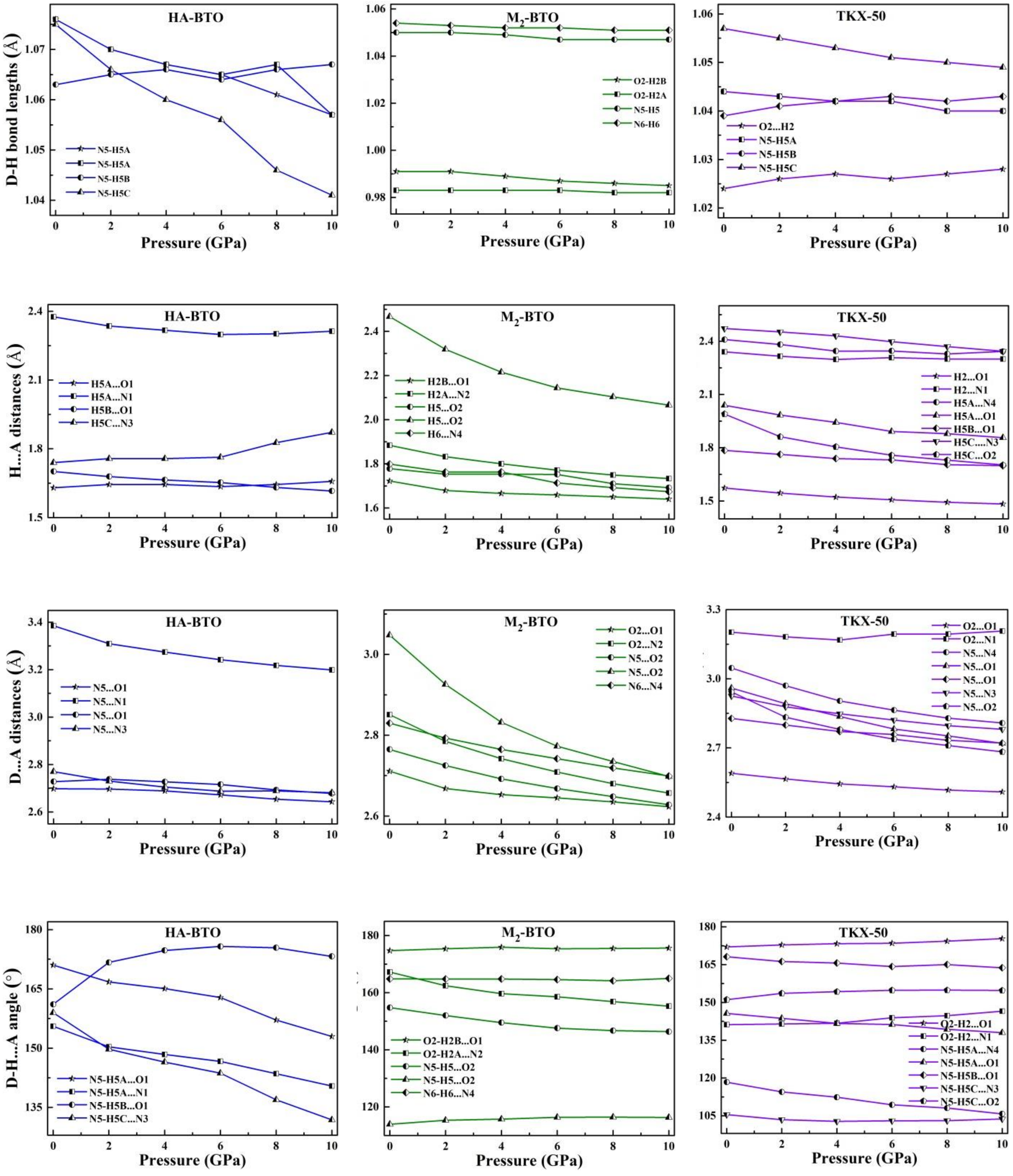

(d) (e) (f)

Fig. S3. Calculated intramolecular D-H bond length, intermolecular D…H and H…A distances, and D-H…A bond angle as a function of pressure for (a) ABTOX, (b) DMA-BTO, (c) DU-BTO, (d) HA-BTO, (e) M_2-BTO and (f) TKX-50.

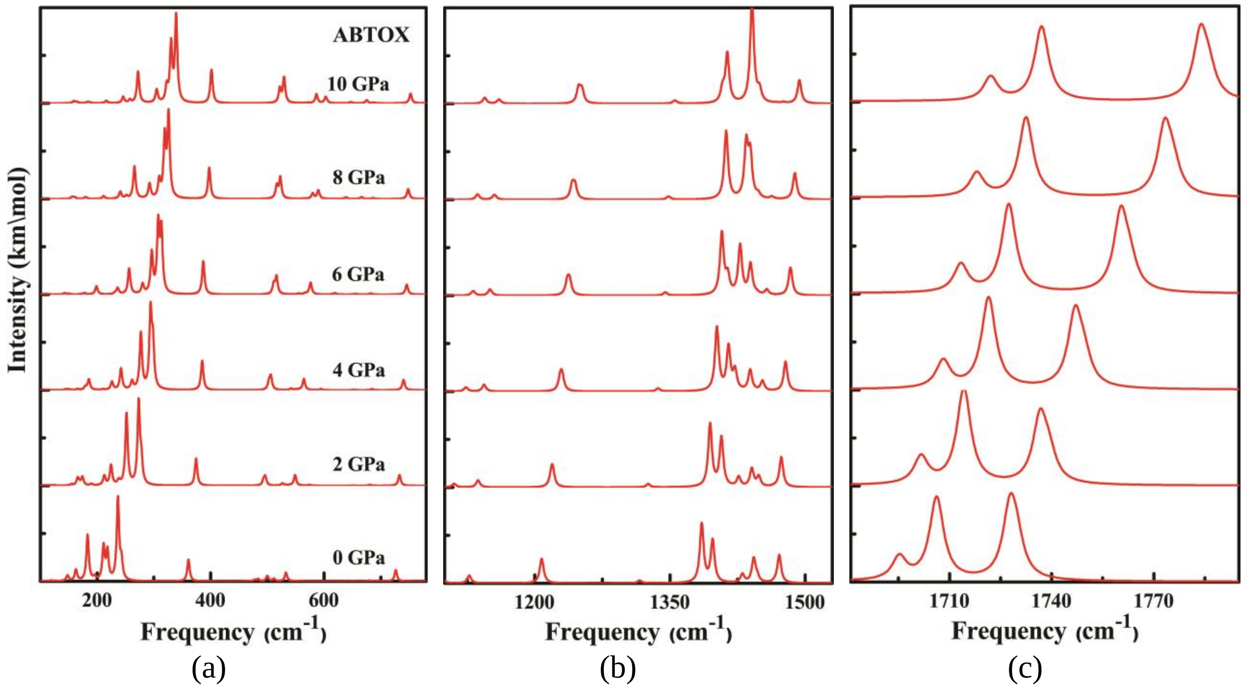

ABTOX
10 GPa
8 GPa
6 GPa
4 GPa
2 GPa
0 GPa
Intensity (km\mol)
200
400
600
1200
1350
1500
1710
1740
1770
Frequency (cm-1)
(a)
(b)
(c)

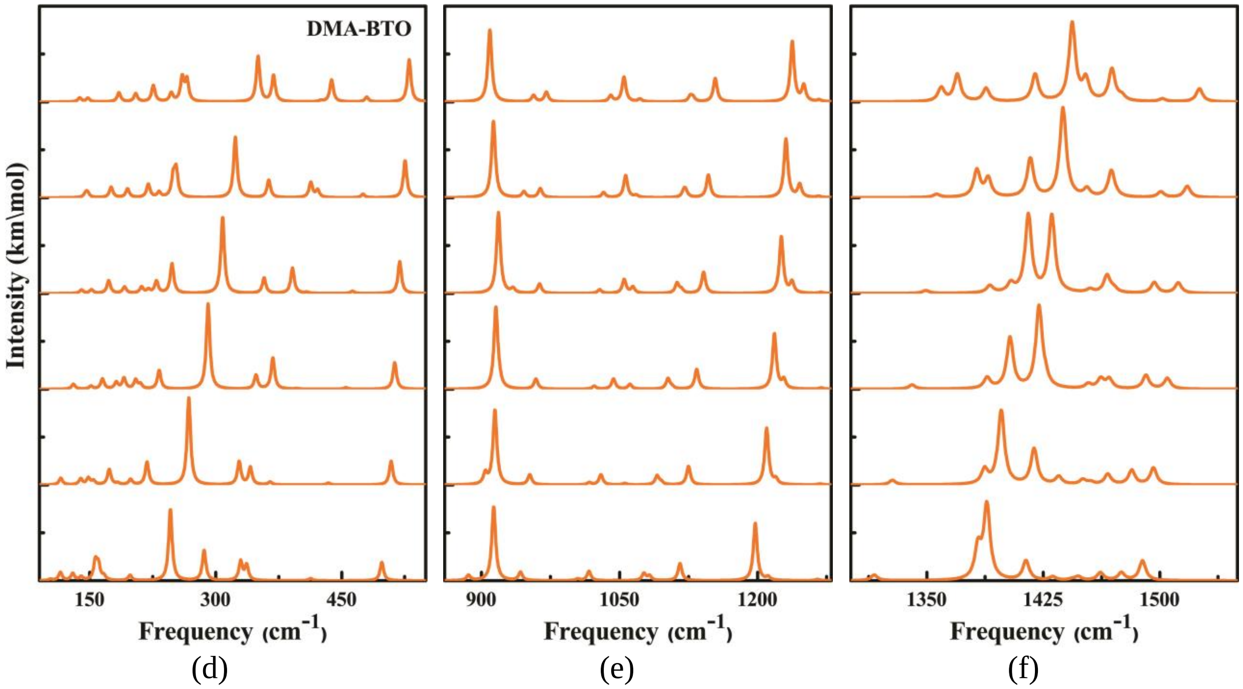

DMA-BTO
Intensity (km\mol)
150
300
450
900
1050
1200
1350
1425
1500
Frequency (cm-1)
(d)
(e)
(f)

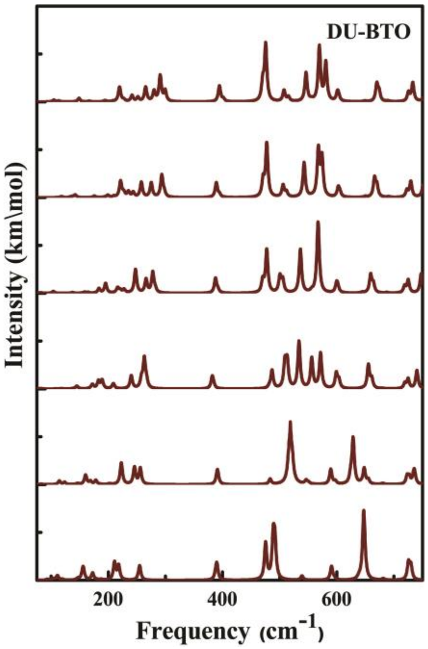
DU-BTO
Intensity (km\mol)
200
400
600
Frequency (cm^{-1})

(g)

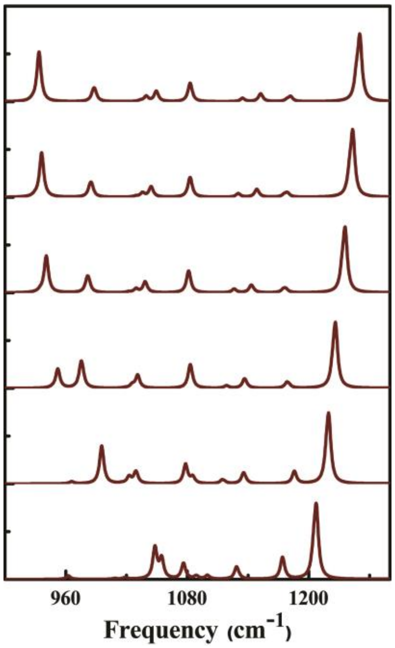
960
1080
1200
Frequency (cm^{-1})

(h)

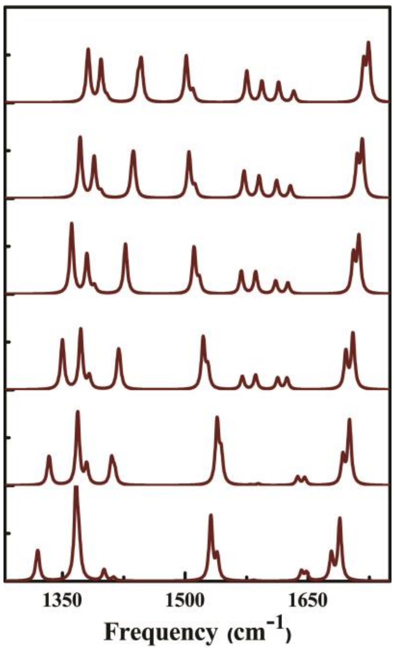
1350
1500
1650
Frequency (cm^{-1})

(i)

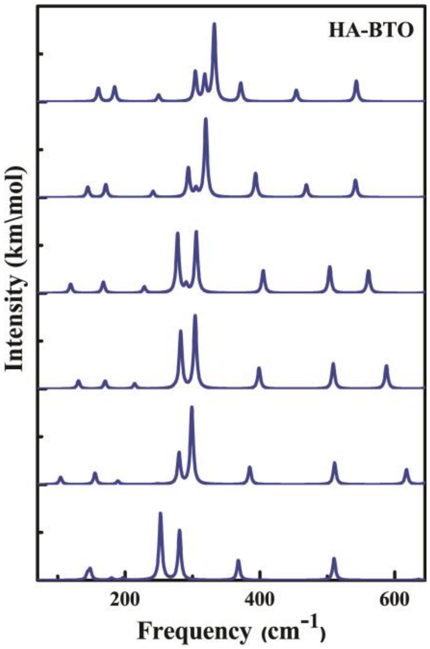
HA-BTO
Intensity (km\mol)
200
400
600
Frequency (cm^{-1})

(j)

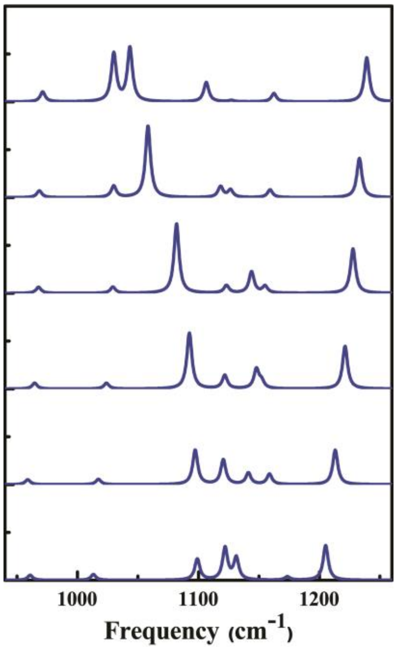
1000
1100
1200
Frequency (cm^{-1})

(k)

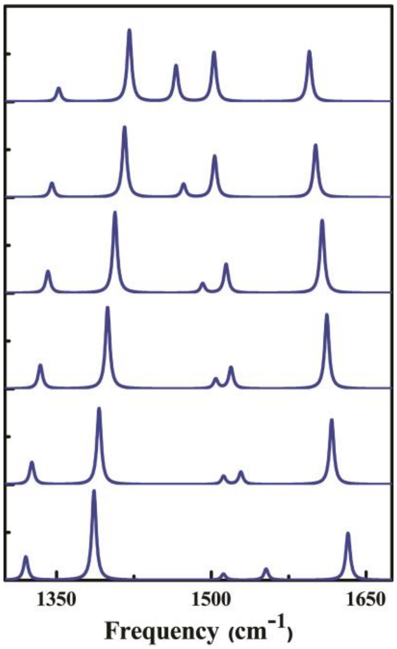
1350
1500
1650
Frequency (cm^{-1})

(l)

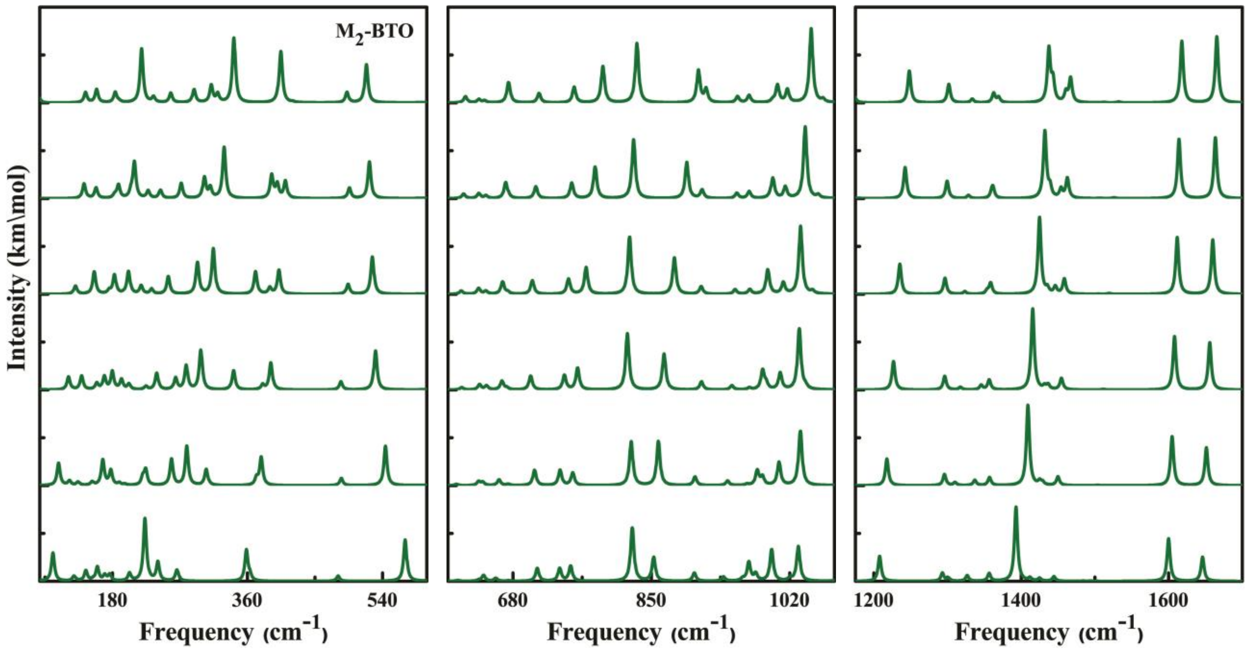

(m) (n) (o)

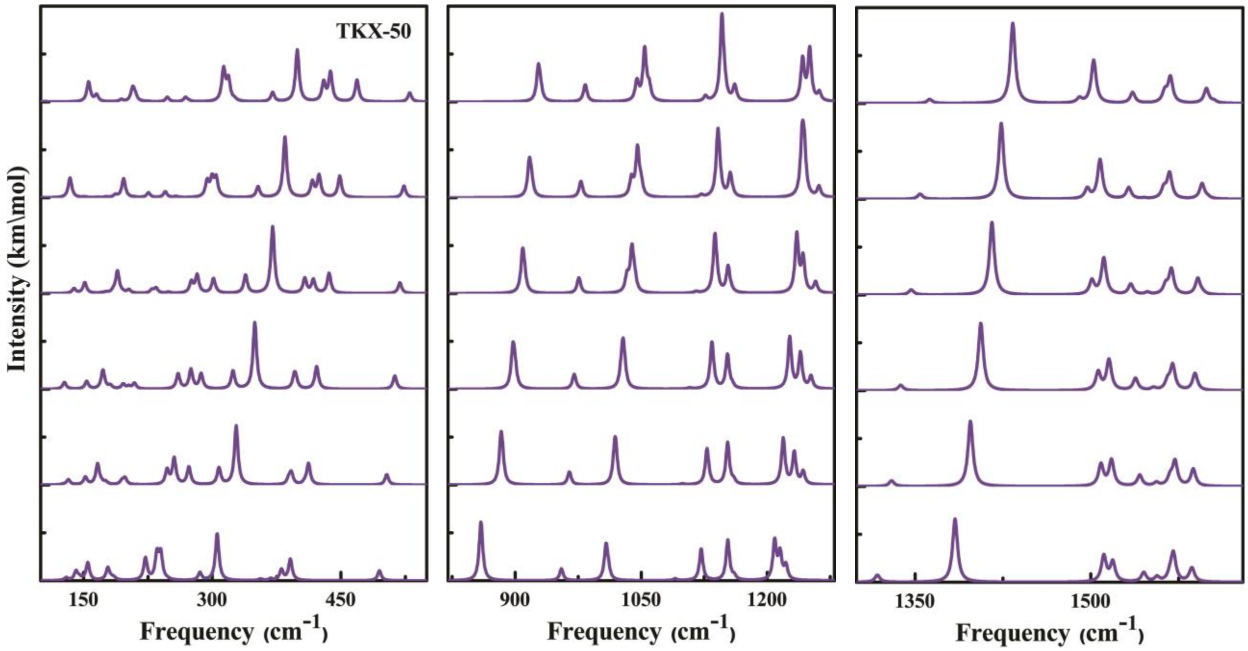

(p) (q) (r)

Fig. S4. Calculated IR spectra of (a-c) ABTO, (d-f) DMA-BTO, (g-i) DU-BTO, (j-l) HA-BTO, (m-o) M_2-BTO and (p-r) TKX-50 without high frequency symmetric/asymmetric stretching vibrations.
